# 3,4-Dihydr­oxy-*N*′-(2-hydroxy­benzyl­idene)benzohydrazide–methanol–water (2/1/3)

**DOI:** 10.1107/S1600536807065038

**Published:** 2007-12-06

**Authors:** Hong-Bo Ma, Shan-Shan Huang, Yun-Peng Diao

**Affiliations:** a Department of Nutrition, Jilin Medical College, Jilin 132013, People’s Republic of China; b School of Pharmacy, Dalian Medical University, Dalian 116044, People’s Republic of China

## Abstract

The asymmetric unit of the title compound, C_14_H_12_N_2_O_4_·0.5CH_4_O·1.5H_2_O, consists of two Schiff base mol­ecules, three water mol­ecules and one methanol mol­ecule. The dihedral angle between the two benzene rings is 7.8 (2)° in one of the mol­ecules and 4.0 (2)° in the other. Intra­molecular O—H⋯O and O—H⋯N hydrogen bonds are observed. Mol­ecules are linked into a three-dimensional network by O—H⋯O and N—H⋯O inter­molecular hydrogen bonds.

## Related literature

For the biological properties of Schiff base compounds, see: Brückner *et al.* (2000 ▶); Harrop *et al.* (2003 ▶); Ren *et al.* (2002 ▶). For related structures, see: Diao (2007 ▶); Diao *et al.* (2007 ▶); Huang *et al.* (2007 ▶); Li *et al.* (2007 ▶).
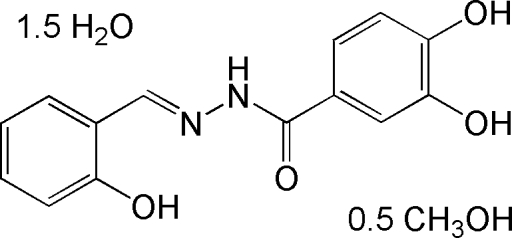


## Experimental

### 

#### Crystal data

C_14_H_12_N_2_O_4_·0.5CH_4_O·1.5H_2_O
*M* *_r_* = 315.30
Triclinic, 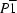

*a* = 10.707 (2) Å
*b* = 11.994 (2) Å
*c* = 14.103 (3) Å
α = 111.56 (3)°
β = 103.13 (3)°
γ = 104.72 (3)°
*V* = 1522.2 (8) Å^3^
*Z* = 4
Mo *K*α radiation
μ = 0.11 mm^−1^
*T* = 298 (2) K
0.17 × 0.15 × 0.15 mm

#### Data collection

Bruker SMART CCD area-detector diffractometer
Absorption correction: multi-scan (*SADABS*; Bruker, 2000 ▶) *T*_min_ = 0.982, *T*_max_ = 0.984
9348 measured reflections
6429 independent reflections
2812 reflections with *I* > 2σ(*I*)
*R*_int_ = 0.030

#### Refinement

*R*[*F* ^2^ > 2σ(*F* ^2^)] = 0.055
*wR*(*F* ^2^) = 0.144
*S* = 0.97
6429 reflections
438 parameters
11 restraints
H atoms treated by a mixture of independent and constrained refinement
Δρ_max_ = 0.17 e Å^−3^
Δρ_min_ = −0.21 e Å^−3^

### 

Data collection: *SMART* (Bruker, 2000 ▶); cell refinement: *SAINT* (Bruker, 2000 ▶); data reduction: *SAINT*; program(s) used to solve structure: *SHELXTL* (Bruker, 2000 ▶); program(s) used to refine structure: *SHELXTL*; molecular graphics: *SHELXTL*; software used to prepare material for publication: *SHELXTL*.

## Supplementary Material

Crystal structure: contains datablocks global, I. DOI: 10.1107/S1600536807065038/ci2538sup1.cif

Structure factors: contains datablocks I. DOI: 10.1107/S1600536807065038/ci2538Isup2.hkl

Additional supplementary materials:  crystallographic information; 3D view; checkCIF report

## Figures and Tables

**Table 1:** Hydrogen-bond geometry (Å, °)

*D*—H⋯*A*	*D*—H	H⋯*A*	*D*⋯*A*	*D*—H⋯*A*
O10—H10*B*⋯O7^i^	0.85 (2)	1.96 (1)	2.793 (3)	168 (3)
O10—H10*A*⋯O6^ii^	0.85 (1)	2.10 (1)	2.938 (3)	173 (3)
O11—H11*A*⋯O8	0.85 (2)	2.09 (1)	2.925 (3)	170 (3)
O12—H12*A*⋯O11^iii^	0.85 (2)	1.94 (1)	2.770 (3)	165 (3)
N1—H1*A*⋯O10	0.91 (3)	1.96 (3)	2.844 (3)	167 (3)
O12—H12*B*⋯O2^iv^	0.85 (2)	2.04 (1)	2.889 (3)	177 (3)
O11—H11*B*⋯O9^v^	0.85 (3)	1.95 (3)	2.765 (3)	162 (3)
N3—H3⋯O12	0.90 (3)	1.96 (3)	2.845 (3)	165 (3)
O8—H8⋯N4	0.82	1.87	2.589 (3)	146
O6—H6⋯O9^vi^	0.82	2.04	2.834 (3)	161
O5—H5⋯O3^vi^	0.82	1.86	2.670 (3)	170
O4—H4⋯N2	0.82	1.84	2.561 (3)	146
O2—H2⋯O1	0.82	2.29	2.725 (3)	114
O2—H2⋯O11^vii^	0.82	1.93	2.703 (3)	158
O1—H1⋯O7^vii^	0.82	1.88	2.695 (3)	172

Symmetry codes: (i) 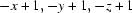; (ii) 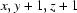; (iii) 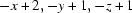; (iv) 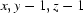; (v) 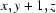; (vi) 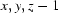; (vii) 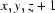.

**Fig. 1.**
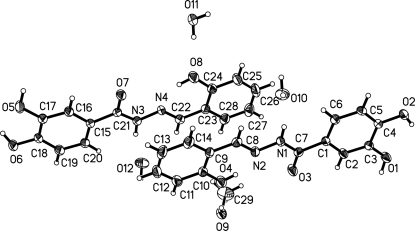
The asymmetric unit of the title compound. Displacement ellipsoids are drawn at the 30% probability level.

**Fig. 2.**
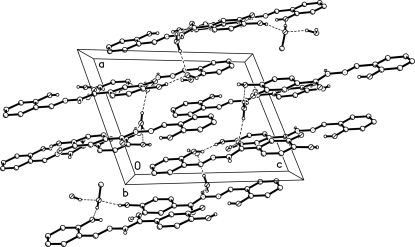
Part of the crystal packing of the title compound.

## supplementary crystallographic information

### Comment

Schiff base compounds have received much attention in recent years. Some of the complexes have been found to have pharmacological and antitumor properties (Brückner *et al.*, 2000; Harrop *et al.*, 2003; Ren *et al.*, 2002). As part of our research programme on the structure of Schiff base compounds (Diao, 2007; Diao *et al.*, 2007; Li *et al.*, 2007; Huang *et al.*, 2007), we report here the structure of the title compound.

The asymmetric unit of the title compound consists of two Schiff base molecules, three water molecules and one methanol molecule (Fig. 1). The corresponding bond lengths and angles in the two Schiff base molecules are nearly identical. The dihedral angle between the C1—C6 and C9—C14 benzene rings is 7.8 (2)° and that between the C15—C20 and C23—C28 benzene rings is 4.0 (2)°. The structure of each molecule is stabilized by O—H···O and O—H···N intramolecular hydrogen bonds.

In the crystal structure, the molecules are linked through intermolecular O—H···O and N—H···O hydrogen bonds (Table 1), forming a three-dimensional network (Fig. 2).

### Experimental

Salicylaldehyde (1.0 mmol, 122.1 mg) and 3,4-dihydroxybenzoic acid hydrazide (1.0 mmol, 168.2 mg) were dissolved in a methanol solution (70 ml). The mixture was stirred at room temperature for 1 h and filtered. After keeping the filtrate in air for a week, yellow block-shaped crystals were formed.

### Refinement

H atoms of the water molecules and –NH groups were located in a difference Fourier map and refined isotropically, with N—H, O—H and H···H (in water) distances restrained to 0.90 (1) Å, 0.85 (1) Å and 1.37 (2) Å, respectively, and with a fixed *U*_iso_ value of 0.08 Å^2^. The remaining H atoms were placed in calculated positions and constrained to ride on their parent atoms, with C—H = 0.93–0.96 Å, O—H = 0.82 Å, and *U*_iso_(H) = 1.2*U*_eq_(C) and 1.5*U*_eq_(O, C_methyl_).

### Crystal data

**Table:**

C_14_H_12_N_2_O_4_·0.5CH_4_O·1.5H_2_O	*Z* = 4
*M**_r_* = 315.30	*F*_000_ = 664
Triclinic, *P*1	*D*_x_ = 1.376 Mg m^−^^3^
Hall symbol: -P 1	Mo *K*α radiation λ = 0.71073 Å
*a* = 10.707 (2) Å	Cell parameters from 1027 reflections
*b* = 11.994 (2) Å	θ = 2.6–24.4º
*c* = 14.103 (3) Å	µ = 0.11 mm^−^^1^
α = 111.56 (3)º	*T* = 298 (2) K
β = 103.13 (3)º	Block, yellow
γ = 104.72 (3)º	0.17 × 0.15 × 0.15 mm
*V* = 1522.2 (8) Å^3^	

### Data collection

**Table:**

Bruker SMART CCD area-detector diffractometer	6429 independent reflections
Radiation source: fine-focus sealed tube	2812 reflections with *I* > 2σ(*I*)
Monochromator: graphite	*R*_int_ = 0.030
*T* = 298(2) K	θ_max_ = 26.9º
ω scans	θ_min_ = 1.7º
Absorption correction: multi-scan(SADABS; Bruker, 2000)	*h* = −13→9
*T*_min_ = 0.982, *T*_max_ = 0.984	*k* = −14→15
9348 measured reflections	*l* = −14→17

### Refinement

**Table:**

Refinement on *F*^2^	Secondary atom site location: difference Fourier map
Least-squares matrix: full	Hydrogen site location: inferred from neighbouring sites
*R*[*F*^2^ > 2σ(*F*^2^)] = 0.055	H atoms treated by a mixture of independent and constrained refinement
*wR*(*F*^2^) = 0.144	*w* = 1/[σ^2^(*F*_o_^2^) + (0.0505*P*)^2^] where *P* = (*F*_o_^2^ + 2*F*_c_^2^)/3
*S* = 0.97	(Δ/σ)_max_ = 0.001
6429 reflections	Δρ_max_ = 0.17 e Å^−^^3^
438 parameters	Δρ_min_ = −0.21 e Å^−^^3^
11 restraints	Extinction correction: none
Primary atom site location: structure-invariant direct methods	

### Special details

**Table:**

Geometry. All e.s.d.'s (except the e.s.d. in the dihedral angle between two l.s. planes) are estimated using the full covariance matrix. The cell e.s.d.'s are taken into account individually in the estimation of e.s.d.'s in distances, angles and torsion angles; correlations between e.s.d.'s in cell parameters are only used when they are defined by crystal symmetry. An approximate (isotropic) treatment of cell e.s.d.'s is used for estimating e.s.d.'s involving l.s. planes.
Refinement. Refinement of *F*^2^ against ALL reflections. The weighted *R*-factor *wR* and goodness of fit *S* are based on *F*^2^, conventional *R*-factors *R* are based on *F*, with *F* set to zero for negative *F*^2^. The threshold expression of *F*^2^ > σ(*F*^2^) is used only for calculating *R*-factors(gt) *etc*. and is not relevant to the choice of reflections for refinement. *R*-factors based on *F*^2^ are statistically about twice as large as those based on *F*, and *R*- factors based on ALL data will be even larger.

### Fractional atomic coordinates and isotropic or equivalent isotropic displacement parameters (Å^2^)

**Table:**

	*x*	*y*	*z*	*U*_iso_*/*U*_eq_	
O1	0.7795 (2)	0.64000 (17)	1.35513 (13)	0.0587 (6)	
H1	0.7625	0.5633	1.3388	0.088*	
O2	0.7267 (2)	0.85547 (16)	1.37077 (14)	0.0528 (5)	
H2	0.7490	0.8323	1.4179	0.079*	
O3	0.7116 (2)	0.39011 (18)	0.96456 (14)	0.0609 (6)	
O4	0.6696 (2)	0.19447 (18)	0.67631 (15)	0.0605 (6)	
H4	0.6723	0.2551	0.7296	0.091*	
O5	0.6555 (2)	0.13711 (18)	−0.10815 (14)	0.0675 (6)	
H5	0.6701	0.2132	−0.0932	0.101*	
O6	0.7472 (2)	−0.05612 (18)	−0.11851 (15)	0.0635 (6)	
H6	0.7496	−0.0212	−0.1586	0.095*	
O7	0.71818 (18)	0.38404 (17)	0.28249 (13)	0.0490 (5)	
O8	0.7971 (2)	0.60616 (18)	0.57457 (15)	0.0625 (6)	
H8	0.7958	0.5464	0.5214	0.094*	
O9	0.8032 (2)	0.04122 (18)	0.73419 (16)	0.0581 (5)	
H9	0.7470	0.0701	0.7128	0.087*	
O10	0.5614 (2)	0.6868 (2)	0.83236 (18)	0.0619 (6)	
O11	0.8239 (2)	0.84887 (19)	0.56226 (15)	0.0563 (6)	
O12	0.9056 (2)	0.11266 (19)	0.42584 (19)	0.0669 (6)	
N1	0.6301 (2)	0.4906 (2)	0.87321 (17)	0.0401 (5)	
N2	0.6220 (2)	0.3958 (2)	0.77792 (17)	0.0406 (5)	
N3	0.8292 (2)	0.30112 (19)	0.37667 (17)	0.0396 (5)	
N4	0.8380 (2)	0.39822 (19)	0.47159 (16)	0.0382 (5)	
C1	0.6883 (2)	0.5837 (2)	1.06912 (19)	0.0370 (6)	
C2	0.7299 (2)	0.5671 (2)	1.16190 (19)	0.0425 (7)	
H2A	0.7506	0.4945	1.1559	0.051*	
C3	0.7415 (3)	0.6543 (2)	1.2623 (2)	0.0404 (6)	
C4	0.7141 (3)	0.7644 (2)	1.2724 (2)	0.0401 (6)	
C5	0.6716 (3)	0.7821 (2)	1.1813 (2)	0.0461 (7)	
H5A	0.6512	0.8549	1.1877	0.055*	
C6	0.6588 (3)	0.6929 (2)	1.0802 (2)	0.0464 (7)	
H6A	0.6303	0.7063	1.0192	0.056*	
C7	0.6776 (3)	0.4813 (3)	0.9654 (2)	0.0418 (7)	
C8	0.5749 (2)	0.4005 (3)	0.6887 (2)	0.0431 (7)	
H8A	0.5451	0.4669	0.6888	0.052*	
C9	0.5675 (2)	0.3036 (3)	0.58771 (19)	0.0399 (6)	
C10	0.6148 (3)	0.2049 (3)	0.5842 (2)	0.0457 (7)	
C11	0.6070 (3)	0.1131 (3)	0.4859 (2)	0.0602 (9)	
H11	0.6400	0.0481	0.4845	0.072*	
C12	0.5506 (3)	0.1181 (3)	0.3907 (3)	0.0722 (11)	
H12	0.5436	0.0554	0.3245	0.087*	
C13	0.5047 (3)	0.2150 (4)	0.3926 (2)	0.0697 (10)	
H13	0.4679	0.2187	0.3280	0.084*	
C14	0.5126 (3)	0.3074 (3)	0.4900 (2)	0.0555 (8)	
H14	0.4809	0.3729	0.4904	0.067*	
C15	0.7553 (3)	0.1977 (2)	0.17858 (19)	0.0389 (6)	
C16	0.7038 (3)	0.2094 (2)	0.0849 (2)	0.0447 (7)	
H16	0.6703	0.2747	0.0890	0.054*	
C17	0.7011 (3)	0.1264 (3)	−0.0143 (2)	0.0475 (7)	
C18	0.7470 (3)	0.0272 (2)	−0.0213 (2)	0.0481 (7)	
C19	0.7935 (3)	0.0113 (3)	0.0699 (2)	0.0581 (8)	
H19	0.8221	−0.0571	0.0645	0.070*	
C20	0.7984 (3)	0.0957 (2)	0.1699 (2)	0.0517 (8)	
H20	0.8306	0.0843	0.2314	0.062*	
C21	0.7657 (3)	0.3000 (2)	0.2826 (2)	0.0385 (6)	
C22	0.8895 (2)	0.3959 (2)	0.5612 (2)	0.0406 (6)	
H22	0.9212	0.3306	0.5616	0.049*	
C23	0.8987 (2)	0.4949 (2)	0.6626 (2)	0.0404 (7)	
C24	0.8529 (3)	0.5961 (3)	0.6665 (2)	0.0462 (7)	
C25	0.8627 (3)	0.6882 (3)	0.7647 (2)	0.0606 (8)	
H25	0.8320	0.7546	0.7667	0.073*	
C26	0.9175 (3)	0.6818 (3)	0.8592 (3)	0.0686 (10)	
H26	0.9241	0.7445	0.9252	0.082*	
C27	0.9630 (3)	0.5839 (3)	0.8583 (2)	0.0653 (9)	
H27	0.9999	0.5801	0.9229	0.078*	
C28	0.9532 (3)	0.4917 (3)	0.7604 (2)	0.0518 (8)	
H28	0.9839	0.4256	0.7596	0.062*	
C29	0.9365 (3)	0.1399 (4)	0.7883 (3)	0.1063 (14)	
H29A	0.9356	0.2106	0.8488	0.159*	
H29B	0.9623	0.1696	0.7384	0.159*	
H29C	1.0019	0.1066	0.8145	0.159*	
H3	0.857 (3)	0.238 (2)	0.382 (2)	0.080*	
H11B	0.799 (3)	0.897 (2)	0.6091 (19)	0.080*	
H12B	0.852 (2)	0.0381 (15)	0.411 (2)	0.080*	
H1A	0.608 (3)	0.5579 (19)	0.871 (2)	0.080*	
H12A	0.9848 (14)	0.110 (3)	0.428 (2)	0.080*	
H11A	0.806 (3)	0.7774 (15)	0.565 (2)	0.080*	
H10A	0.613 (2)	0.7585 (17)	0.840 (2)	0.080*	
H10B	0.4781 (12)	0.677 (3)	0.804 (2)	0.080*	

### Atomic displacement parameters (Å^2^)

**Table:**

	*U*^11^	*U*^22^	*U*^33^	*U*^12^	*U*^13^	*U*^23^
O1	0.0975 (16)	0.0432 (11)	0.0340 (11)	0.0319 (12)	0.0162 (10)	0.0165 (9)
O2	0.0792 (14)	0.0403 (11)	0.0418 (11)	0.0273 (10)	0.0260 (11)	0.0157 (9)
O3	0.1012 (16)	0.0528 (13)	0.0427 (12)	0.0477 (12)	0.0267 (11)	0.0225 (10)
O4	0.0862 (15)	0.0518 (13)	0.0495 (13)	0.0376 (12)	0.0249 (12)	0.0205 (11)
O5	0.1172 (18)	0.0582 (13)	0.0381 (12)	0.0494 (14)	0.0273 (11)	0.0220 (10)
O6	0.1113 (17)	0.0439 (12)	0.0450 (12)	0.0406 (12)	0.0357 (12)	0.0174 (10)
O7	0.0684 (13)	0.0426 (11)	0.0432 (11)	0.0319 (10)	0.0213 (9)	0.0185 (9)
O8	0.0940 (16)	0.0526 (13)	0.0508 (13)	0.0424 (12)	0.0278 (12)	0.0222 (11)
O9	0.0649 (14)	0.0559 (13)	0.0523 (13)	0.0294 (11)	0.0210 (11)	0.0185 (10)
O10	0.0650 (14)	0.0575 (14)	0.0714 (15)	0.0288 (12)	0.0174 (13)	0.0375 (13)
O11	0.0738 (14)	0.0496 (13)	0.0485 (13)	0.0278 (12)	0.0303 (10)	0.0168 (11)
O12	0.0593 (14)	0.0522 (13)	0.0956 (16)	0.0269 (12)	0.0253 (14)	0.0371 (13)
N1	0.0532 (14)	0.0373 (13)	0.0330 (13)	0.0209 (11)	0.0183 (11)	0.0146 (11)
N2	0.0461 (14)	0.0429 (13)	0.0334 (13)	0.0182 (11)	0.0158 (10)	0.0157 (11)
N3	0.0494 (14)	0.0353 (13)	0.0382 (13)	0.0191 (11)	0.0202 (11)	0.0157 (11)
N4	0.0444 (13)	0.0343 (12)	0.0326 (13)	0.0145 (10)	0.0158 (10)	0.0107 (10)
C1	0.0433 (16)	0.0346 (15)	0.0330 (15)	0.0160 (12)	0.0144 (12)	0.0139 (12)
C2	0.0541 (17)	0.0385 (15)	0.0367 (16)	0.0246 (14)	0.0139 (13)	0.0155 (13)
C3	0.0493 (17)	0.0365 (16)	0.0343 (15)	0.0152 (13)	0.0132 (13)	0.0164 (13)
C4	0.0484 (17)	0.0315 (15)	0.0395 (16)	0.0143 (13)	0.0195 (13)	0.0133 (13)
C5	0.0668 (19)	0.0341 (16)	0.0479 (17)	0.0263 (14)	0.0255 (15)	0.0213 (14)
C6	0.0663 (19)	0.0428 (17)	0.0372 (16)	0.0245 (15)	0.0196 (14)	0.0218 (14)
C7	0.0511 (17)	0.0421 (17)	0.0423 (17)	0.0221 (14)	0.0210 (13)	0.0236 (14)
C8	0.0419 (16)	0.0460 (17)	0.0465 (17)	0.0165 (13)	0.0189 (13)	0.0240 (15)
C9	0.0363 (15)	0.0448 (17)	0.0320 (15)	0.0082 (13)	0.0118 (12)	0.0154 (13)
C10	0.0476 (17)	0.0429 (17)	0.0414 (17)	0.0103 (14)	0.0208 (14)	0.0150 (14)
C11	0.066 (2)	0.0478 (19)	0.059 (2)	0.0121 (16)	0.0345 (17)	0.0141 (16)
C12	0.074 (2)	0.060 (2)	0.043 (2)	−0.0081 (19)	0.0279 (18)	0.0009 (18)
C13	0.063 (2)	0.083 (3)	0.0344 (19)	−0.005 (2)	0.0102 (15)	0.0223 (19)
C14	0.0486 (18)	0.067 (2)	0.0442 (18)	0.0118 (16)	0.0135 (15)	0.0269 (17)
C15	0.0498 (16)	0.0331 (15)	0.0353 (15)	0.0158 (13)	0.0191 (13)	0.0144 (13)
C16	0.0624 (19)	0.0397 (16)	0.0397 (16)	0.0274 (14)	0.0214 (14)	0.0182 (14)
C17	0.066 (2)	0.0400 (17)	0.0391 (17)	0.0231 (15)	0.0198 (15)	0.0183 (14)
C18	0.072 (2)	0.0326 (16)	0.0368 (16)	0.0193 (15)	0.0221 (14)	0.0108 (13)
C19	0.094 (2)	0.0401 (17)	0.0498 (19)	0.0364 (17)	0.0301 (17)	0.0202 (15)
C20	0.082 (2)	0.0402 (17)	0.0418 (17)	0.0300 (16)	0.0247 (15)	0.0211 (14)
C21	0.0483 (17)	0.0343 (15)	0.0379 (16)	0.0162 (13)	0.0199 (13)	0.0181 (13)
C22	0.0368 (15)	0.0391 (16)	0.0443 (17)	0.0139 (13)	0.0146 (13)	0.0172 (14)
C23	0.0358 (15)	0.0408 (16)	0.0365 (16)	0.0082 (13)	0.0110 (12)	0.0143 (13)
C24	0.0454 (17)	0.0419 (17)	0.0424 (17)	0.0091 (14)	0.0170 (13)	0.0139 (14)
C25	0.068 (2)	0.0479 (19)	0.052 (2)	0.0184 (16)	0.0275 (17)	0.0067 (16)
C26	0.064 (2)	0.064 (2)	0.043 (2)	0.0083 (19)	0.0207 (17)	−0.0015 (17)
C27	0.056 (2)	0.082 (3)	0.0359 (18)	0.0100 (19)	0.0089 (15)	0.0192 (18)
C28	0.0489 (18)	0.0583 (19)	0.0454 (18)	0.0160 (15)	0.0149 (14)	0.0245 (16)
C29	0.066 (3)	0.096 (3)	0.126 (3)	0.015 (2)	0.010 (2)	0.043 (3)

### Geometric parameters (Å, °)

**Table:**

O1—C3	1.367 (3)	C6—H6A	0.93
O1—H1	0.82	C8—C9	1.440 (3)
O2—C4	1.365 (3)	C8—H8A	0.93
O2—H2	0.82	C9—C10	1.390 (4)
O3—C7	1.234 (3)	C9—C14	1.392 (3)
O4—C10	1.363 (3)	C10—C11	1.386 (4)
O4—H4	0.82	C11—C12	1.372 (4)
O5—C17	1.368 (3)	C11—H11	0.93
O5—H5	0.82	C12—C13	1.365 (4)
O6—C18	1.368 (3)	C12—H12	0.93
O6—H6	0.82	C13—C14	1.380 (4)
O7—C21	1.240 (3)	C13—H13	0.93
O8—C24	1.361 (3)	C14—H14	0.93
O8—H8	0.82	C15—C16	1.384 (3)
O9—C29	1.416 (3)	C15—C20	1.388 (3)
O9—H9	0.82	C15—C21	1.484 (3)
O10—H10A	0.846 (10)	C16—C17	1.375 (3)
O10—H10B	0.85 (2)	C16—H16	0.93
O11—H11B	0.85 (3)	C17—C18	1.378 (4)
O11—H11A	0.85 (2)	C18—C19	1.370 (4)
O12—H12B	0.85 (2)	C19—C20	1.381 (4)
O12—H12A	0.85 (2)	C19—H19	0.93
N1—C7	1.341 (3)	C20—H20	0.93
N1—N2	1.372 (3)	C22—C23	1.447 (3)
N1—H1A	0.91 (3)	C22—H22	0.93
N2—C8	1.272 (3)	C23—C28	1.388 (3)
N3—C21	1.340 (3)	C23—C24	1.407 (4)
N3—N4	1.378 (3)	C24—C25	1.379 (4)
N3—H3	0.90 (3)	C25—C26	1.367 (4)
N4—C22	1.273 (3)	C25—H25	0.93
C1—C6	1.387 (3)	C26—C27	1.379 (4)
C1—C2	1.387 (3)	C26—H26	0.93
C1—C7	1.481 (3)	C27—C28	1.376 (4)
C2—C3	1.372 (3)	C27—H27	0.93
C2—H2A	0.93	C28—H28	0.93
C3—C4	1.388 (3)	C29—H29A	0.96
C4—C5	1.373 (3)	C29—H29B	0.96
C5—C6	1.383 (3)	C29—H29C	0.96
C5—H5A	0.93		
			
C3—O1—H1	109.5	C12—C13—C14	120.4 (3)
C4—O2—H2	109.5	C12—C13—H13	119.8
C10—O4—H4	109.5	C14—C13—H13	119.8
C17—O5—H5	109.5	C13—C14—C9	120.6 (3)
C18—O6—H6	109.5	C13—C14—H14	119.7
C24—O8—H8	109.5	C9—C14—H14	119.7
C29—O9—H9	109.5	C16—C15—C20	118.5 (2)
H10A—O10—H10B	108 (2)	C16—C15—C21	116.7 (2)
H11B—O11—H11A	107 (2)	C20—C15—C21	124.7 (2)
H12B—O12—H12A	108 (2)	C17—C16—C15	121.4 (3)
C7—N1—N2	117.5 (2)	C17—C16—H16	119.3
C7—N1—H1A	123.7 (19)	C15—C16—H16	119.3
N2—N1—H1A	118.7 (19)	O5—C17—C16	123.3 (3)
C8—N2—N1	119.1 (2)	O5—C17—C18	117.2 (2)
C21—N3—N4	117.2 (2)	C16—C17—C18	119.5 (3)
C21—N3—H3	124.4 (19)	O6—C18—C19	119.2 (3)
N4—N3—H3	118.0 (18)	O6—C18—C17	120.9 (2)
C22—N4—N3	118.1 (2)	C19—C18—C17	119.9 (3)
C6—C1—C2	118.0 (2)	C18—C19—C20	120.8 (3)
C6—C1—C7	125.4 (2)	C18—C19—H19	119.6
C2—C1—C7	116.6 (2)	C20—C19—H19	119.6
C3—C2—C1	122.0 (3)	C19—C20—C15	119.9 (3)
C3—C2—H2A	119.0	C19—C20—H20	120.0
C1—C2—H2A	119.0	C15—C20—H20	120.0
O1—C3—C2	123.7 (2)	O7—C21—N3	120.5 (2)
O1—C3—C4	117.0 (2)	O7—C21—C15	120.6 (2)
C2—C3—C4	119.3 (2)	N3—C21—C15	118.9 (2)
O2—C4—C5	118.8 (2)	N4—C22—C23	119.9 (3)
O2—C4—C3	121.6 (2)	N4—C22—H22	120.1
C5—C4—C3	119.6 (2)	C23—C22—H22	120.1
C4—C5—C6	120.7 (3)	C28—C23—C24	117.7 (3)
C4—C5—H5A	119.7	C28—C23—C22	120.0 (3)
C6—C5—H5A	119.7	C24—C23—C22	122.3 (2)
C5—C6—C1	120.4 (2)	O8—C24—C25	117.9 (3)
C5—C6—H6A	119.8	O8—C24—C23	121.7 (2)
C1—C6—H6A	119.8	C25—C24—C23	120.4 (3)
O3—C7—N1	121.3 (2)	C26—C25—C24	120.0 (3)
O3—C7—C1	120.6 (2)	C26—C25—H25	120.0
N1—C7—C1	118.1 (2)	C24—C25—H25	120.0
N2—C8—C9	120.3 (3)	C25—C26—C27	121.0 (3)
N2—C8—H8A	119.8	C25—C26—H26	119.5
C9—C8—H8A	119.8	C27—C26—H26	119.5
C10—C9—C14	118.2 (3)	C28—C27—C26	119.1 (3)
C10—C9—C8	121.8 (2)	C28—C27—H27	120.4
C14—C9—C8	120.0 (3)	C26—C27—H27	120.4
O4—C10—C11	117.7 (3)	C27—C28—C23	121.7 (3)
O4—C10—C9	121.7 (2)	C27—C28—H28	119.2
C11—C10—C9	120.6 (3)	C23—C28—H28	119.2
C12—C11—C10	120.0 (3)	O9—C29—H29A	109.5
C12—C11—H11	120.0	O9—C29—H29B	109.5
C10—C11—H11	120.0	H29A—C29—H29B	109.5
C13—C12—C11	120.2 (3)	O9—C29—H29C	109.5
C13—C12—H12	119.9	H29A—C29—H29C	109.5
C11—C12—H12	119.9	H29B—C29—H29C	109.5

### Hydrogen-bond geometry (Å, °)

**Table:**

*D*—H···*A*	*D*—H	H···*A*	*D*···*A*	*D*—H···*A*
O10—H10B···O7^i^	0.85 (2)	1.96 (1)	2.793 (3)	168 (3)
O10—H10A···O6^ii^	0.85 (1)	2.10 (1)	2.938 (3)	173 (3)
O11—H11A···O8	0.85 (2)	2.09 (1)	2.925 (3)	170 (3)
O12—H12A···O11^iii^	0.85 (2)	1.94 (1)	2.770 (3)	165 (3)
N1—H1A···O10	0.91 (3)	1.96 (3)	2.844 (3)	167 (3)
O12—H12B···O2^iv^	0.85 (2)	2.04 (1)	2.889 (3)	177 (3)
O11—H11B···O9^v^	0.85 (3)	1.95 (3)	2.765 (3)	162 (3)
N3—H3···O12	0.90 (3)	1.96 (3)	2.845 (3)	165 (3)
O8—H8···N4	0.82	1.87	2.589 (3)	146
O6—H6···O9^vi^	0.82	2.04	2.834 (3)	161
O5—H5···O3^vi^	0.82	1.86	2.670 (3)	170
O4—H4···N2	0.82	1.84	2.561 (3)	146
O2—H2···O1	0.82	2.29	2.725 (3)	114
O2—H2···O11^vii^	0.82	1.93	2.703 (3)	158
O1—H1···O7^vii^	0.82	1.88	2.695 (3)	172

Symmetry codes: (i) −*x*+1, −*y*+1, −*z*+1; (ii) *x*, *y*+1, *z*+1; (iii) −*x*+2, −*y*+1, −*z*+1; (iv) *x*, *y*−1, *z*−1; (v) *x*, *y*+1, *z*; (vi) *x*, *y*, *z*−1; (vii) *x*, *y*, *z*+1.
